# Understanding how birds rebuild fat stores during migration: insights from an experimental study

**DOI:** 10.1038/s41598-019-46487-z

**Published:** 2019-07-11

**Authors:** Pedro M. Araújo, Ivan Viegas, Afonso D. Rocha, Auxiliadora Villegas, John G. Jones, Liliana Mendonça, Jaime A. Ramos, José A. Masero, José A. Alves

**Affiliations:** 1 MARE – Marine and Environmental Sciences Centre, Department of Life Sciences, University of Coimbra, 3004-517 Coimbra, Portugal; 2 CIBIO/InBIO, Centro de Investigação em Biodiversidade e Recursos Genéticos, Universidade do Porto, 4485-661 Vairão, Portugal; 3 CNC - Center for Neuroscience and Cell Biology, University of Coimbra, Coimbra, 3004- 517 Portugal; 4 CEF - Center for Functional Ecology, Department Life Sciences, University of Coimbra, Coimbra, 3000-456 Portugal; 5 Department of Biology & CESAM, University of Aveiro, Campus Universitário de Santiago, 3180-193 Aveiro, Portugal; 6 Conservation Biology Research Group, Área de Zoología, Universidad de Extremadura, Avenida de Elvas s/n, 06071 Badajoz, Spain; 7 University of Iceland, South Iceland Research Centre, Lindarbraut 4, IS-840 Laugarvatn, Iceland

**Keywords:** Animal migration, Animal migration, Ecophysiology, Ecophysiology

## Abstract

Mechanisms underlying fat accumulation for long-distance migration are not fully understood. This is especially relevant in the context of global change, as many migrants are dealing with changes in natural habitats and associated food sources and energy stores. The continental Black-tailed godwit *Limosa limosa limosa* is a long-distance migratory bird that has undergone a considerable dietary shift over the past few decades. Historically, godwits fed on an animal-based diet, but currently, during the non-breeding period godwits feed almost exclusively on rice seeds. The latter diet may allow building up of their fuel stores for migration by significantly increasing *de novo* lipogenesis (*DNL*) activity. Here, we performed an experiment to investigate lipid flux and the abundance of key enzymes involved in *DNL* in godwits, during fasting and refueling periods at the staging site, while feeding on rice seeds or fly larvae. Despite no significant differences found in enzymatic abundance (*FASN*, *ME1*, *ACC* and *LPL*) in stored fat, experimental godwits feeding on rice seeds presented high rates of *DNL* when compared to fly-larvae fed birds (~35 times more) and fasted godwits (no *DNL* activity). The increase of fractional *DNL* in godwits feeding on a carbohydrate-rich diet can potentially be enhanced by the fasting period that stimulates lipogenesis. Although requiring further testing, these recent findings provide new insights into the mechanisms of avian fat accumulation during a fasting and refueling cycle and associated responses to habitat and dietary changes in a migratory species.

## Introduction

Migratory animals need to adjust their physiology to accumulate high amounts of fat in preparation for migratory journeys^1^. In these species, the ability to store and mobilize fat stores efficiently is essential^2^. During the migratory cycle, birds experience different physiological states and fat compounds are mobilized (fasting) or stored (fueling or refueling)^3,4^. Migratory movements are mainly fuelled by the oxidation of fatty acids (FA) stored in the form of energy-rich triglycerides (TAG) in adipose tissue, which is delivered to muscles by the circulatory system^3–5^. Long-distance migration between breeding grounds, stopovers and non-breeding grounds is typically carried out in cycles of refueling at stopover areas and fasting while in flight or at stopover sites^3^. During migratory flights, birds require high fat stores acquired at staging areas^6^, and the ability to manage their energy supply constraints by using stores acquired at stopovers is crucial. Birds during migratory flight or induced fasting showed several similarities in terms of used energy sources, but showed different energy turnover rates, which is significantly higher in flying birds^7^. However, fasting can be used to simulate migratory flight if fasting time is not long enough to produce changes in body mass composition^7^. Most migrants currently face severe and rapid challenges regarding access to their traditional food sources, with many species increasingly using anthropogenic habitats with associated changes in their diet^8–10^. The physiological and metabolic adaptations involved in such dietary shifts regarding lipid accumulation and synthesis, in preparation for migratory flights, remain mostly unclear despite its fundamental role in the life-cycle of avian migrants.

Recent evidence highlighted that not only the amount of fat stored but also FA composition influence bird migratory performance (reviewed^11^). FA chain length, degree of unsaturation, and placement of double bonds can all affect the rate of mobilization, transport, and oxidation of lipid reserves during flight, thus influencing overall flight endurance (e.g.^12,13^). Furthermore, higher levels of free fatty acids (FFA) and glycerol in plasma results from the catabolism of fat stores in adipose tissue^5^. Although birds can alter the FA composition of their fat stores and the phospholipids in membranes through endogenous mechanisms or through diet^12^, they are unable to synthesize several important polyunsaturated FA (PUFAs) such as omega-3 and omega-6, which must be strictly derived from dietary sources^12^. Studies based on migrating waterbirds performing long non-stop flights have proposed that omega-3 PUFAs play a significant role in migratory performance by enhancing the aerobic capacity of flight muscles^13^. Migratory birds should therefore strive to include as much non-synthesisable PUFA as possible in their diet (the natural doping hypothesis)^13^. Some studies support the natural doping hypothesis as observed in refueling migratory semipalmated sandpipers (*Calidris pusilla*) and in captive sedentary bobwhite quail (*Collinus virginianus*) that feed on a PUFA (e.g. DHA and EPA) rich diet, where the regulation of metabolic enzyme activity was influenced by the dietary FA^14–16^. However, Price and Guglielmo^12^ compared n-3 PUFA and n-6 PUFA diets in migratory white-throated sparrows *Zonotrichia albicollis*, and no correlations were found between either aerobic and oxidative enzymes, or DHA and EPA PUFAS in flight muscle phospholipids. These data indicate that upregulation of aerobic capacity is not diet-controlled, or that n-3 and n-6 PUFA can show a similar or no effect. Furthermore, yellow-rumped warblers *Setophaga coronate* supplemented with n-3 PUFA showed no effect of PUFA on their flight performance, contradicting the natural doping hypothesis^17,18^. Overall, some studies did not support the natural doping hypothesis, or the PUFA benefits on migratory performance^12,17,18^.

Long distance flights demand intense muscular activity during a period of fasting, which may enhance the need to quickly re-fuel. According to Ramenofsky^19^, after a fasting period, lipogenesis increases rapidly and occurs primarily in the liver^20,21^, with several enzymes likely being crucial to this process both in the liver and in the adipose tissue. The acetyl-CoA carboxylase (*ACC*) and fatty acid synthetase (*FASN*) are the main enzyme complexes involved in lipogenesis^10^. *ACC* is considered a key enzyme for lipogenesis as it promotes FA synthesis at high rates^22–24^, while *FASN* controls FA synthesis in lower lipogenesis rates^10^. Lipoprotein lipase (*LPL*) activity is also important in lipid metabolism and this enzyme is known to be strictly regulated during the migratory cycle, mainly during early stages of refueling^19^. LPL’s catalytic activity in the hydrolysis of triglycerides, on both muscle and adipose tissues, provides FFA resulting in fat storage. Some studies assumed that malic enzyme (*ME1*) is not involved in FA synthesis^24^, but Shah *et al.*^25^ found that *ME1* activity increased in the liver during the pre-migratory phase. These enzymes may play a significant role in lipogenesis during migration, and therefore, assessing their expression patterns can help understand the metabolic pathways at play during fuelling and fasting cycles, which might differ according to dietary sources.

Most shorebirds, specifically those that undertake long migrations by continuous powered flight, present a high capacity to store and rapidly use adipose fat on a strict annual schedule^26–28^, with many using stopover or staging sites to refuel between flights along their routes^29^. The black-tailed godwit *Limosa limosa* (henceforth, godwit) is a long-distance migrant that traditionally foraged in natural wetlands, where it fed mainly on animal prey^30,31^. However, in recent decades it has dramatically changed its feeding habits throughout its non-breeding period, particularly the Western European population (*Limosa limosa limosa*), relying on rice fields located in Iberia and West Africa^32,33^, where they feed on unharvested rice seeds. This population, therefore, is increasingly dependent on artificial wetlands throughout its non-breeding range, most of which are rice fields located in Iberia and West Africa^33,34^. Using deuterated water (^2^H2O) as a tracer for *de novo* lipogenesis (*DNL*), a recent study demonstrated the synthesis of FA from non-lipid precursors (such as carbohydrates), showing that godwits are able to increase their *DNL* rate when they feed on a poor lipidic dietary during the winter period^34^.

In the present study, we mimicked a re-fuelling event using two different diets sources: fly larvae (traditional animal-based diet – 20% fat, including omega-3) and rice seeds (current plant-based diet − 2% fat and omega-3 free) in order to examine how dietary conditions may influence *DNL* rates. Using this approach, we aimed to understand whether the *DNL* in a migratory shorebird varies according to diet conditions. Furthermore, we measured the mRNA abundance in order to understand whether the godwits used different metabolic pathways during the re-fuelling phase, given the two different diets sources.

## Methods

### Bird capture and experimental setup

**Figure 1:**
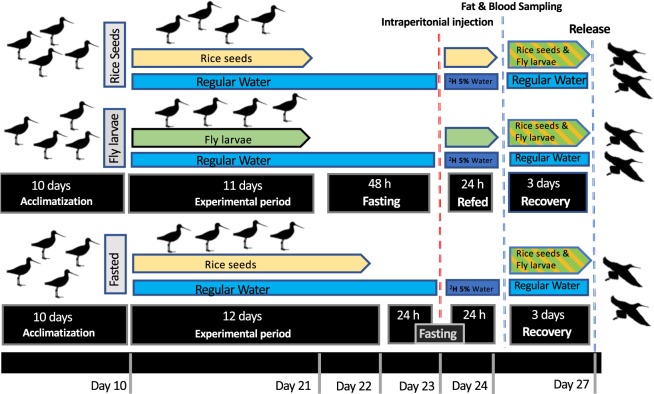
Schematic representation of experimental design from the capture of black-tailed godwits in the Extremadura rice fields onwards (Badajoz, Spain).

Migrating godwits traveling from West Africa to the breeding grounds in western Europe were captured (n = 23) with mist nets in January of 2018 at the Extremadura rice fields (Southwest Spain 39°02^’^ N, 5°56’ W^31^), an important stopover site for this population. Godwits were measured, ringed and transported to certified avian facilities at the University of Extremadura (Badajoz, Spain) where they were randomly split into six separate outdoor aviaries (5 × 2.5 × 2 m each, maximum four birds per cage). Acclimation to conditions in captivity lasted for 10 days with *ad libitum* access to water for drinking and bathing and unprocessed rice seeds from Extremadura rice fields. Food was provided in trays with water which were changed twice daily. After demonstrating no signs of behavioural distress (e.g. increased respiratory rate or non-responsiveness to external stimuli) and sustaining a steady consumption of the food available in the trays for several days, symptomatic of a stabilised acclimation to captivity, the experimental procedures were initiated (Fig. 1).

Individuals were randomly assigned to one of three study groups: Rice group (n = 8), Larvae group (n = 8) and Fasted group (n = 7). Rice and Larvae groups were fed on unprocessed rice seeds and fly larvae (*Protophormia terraenovae*), respectively, until increasing feeding activity, which was indicative of and assumed as entering the refueling phase. The Fasted group was fed with unprocessed rice seeds (godwits staging at Extremadura feed mostly on rice seeds^10^, and it acted as a control group in relation to re-refueling (i.e. this group was sampled during the fasting phase prior to refueling, Fig. 1). Both diets, rice seeds and fly larvae, differed significantly in the nutritional composition and energy density: unprocessed rice seeds consist mainly of starch and fiber, with less moisture and lower energetic density than fly larvae, which contain mostly protein^34^. Furthermore, the fly larvae diet has a lipid content 10 times greater than unprocessed rice seeds, and it includes PUFAs^34^. Each group was subjected to the dietary condition during the following 11–12 days.

The Fasted group was deprived of food for 24 h, injected with ^2^H_2_O, then fasted for another 24 h. The Rice and Larvae groups were deprived of food for 48 h, injected with ^2^H_2_O and then allowed to feed for 24 h with access to the previously assigned diet. The ^2^H_2_O was delivered via intraperitoneal injection (see details in^34^) with 99.8%-enriched ^2^H_2_O (CortecNet, France; 5% volume per gram body mass), and 0.9% saline. After injection, all groups were supplied with 5%-enriched drinking ^2^H_2_O to maintain body water ^2^H-enrichment. All birds were sampled in early morning, 24 h post-injection for blood and visible subcutaneous fat according to Rocha *et al*.^35^. No godwit was kept for more than 48 h in fasting (Fig. 1). The birds recovered immediately after adipose tissue sampling but were kept under observation for the following three days, with food and water provided *ad libitum*. No alterations of behavior or feeding were observed during this period, and all birds were successfully released into the wild at the original capture site (Fig. 1).

### Sample collection and processing

Blood was collected from the brachial vein (150 μl), pierced with a 26-gauge needle, collected into a heparinized capillary tube (Microvette CB 300; Sarstedt AG & Co., Germany), centrifuged at 10.062 G for 10 min and stored at −20 °C until further analysis. Quantifications of plasma glucose, TAG and cholesterol were performed in a fully-automated analyzer Miura 200 (I.S.E. S.r.l.; Guidonia, Italy) using its dedicated reagent kits (ref. A-R0100000601, A-R0100000901, and R0100000501 respectively; n = 22). Body water ^2^H-enrichments were determined by nuclear magnetic resonance (NMR) analysis, using 10 μl of plasma analyzed in duplicate^36^, where water content was assumed to be 92% of total plasma (n = 21).

We performed subcutaneous fat biopsies (see above) for TAG analysis (16.6 ± 4.74 mg, n = 22) and for mRNA abundance (9.7 ± 4.89 mg, n = 23). Fat samples for NMR analysis were kept in methyl tert-butyl ether (MTBE; Sigma, Spain); TAG was extracted according to^37^ and stored at −20 °C until analysis. Despite the methodology being adapted for recovery of most lipid classes^37^, it was assumed that the majority of lipids in subcutaneous fat would be storage neutral lipids (~90%), mainly TAG^38^. A methodological control for TAG recovery was performed (see below FA/glycerol ratio). NMR spectra of TAG samples were obtained at 25 °C with a Bruker Avance III HD system with an UltraShield Plus magnet (11.7 T, ^1^H operating frequency 500 MHz) and equipped with a 5-mm ^2^H-selective probe with ^19^F lock and ^1^H-decoupling coil. NMR spectra were analyzed using the curve-fitting routine supplied with ACD Labs 1D NMR processor software 2.4^34^. As control for the TAG extraction, a FA/glycerol ratio was calculated from the area of all FA α protons times two, divided by TAG-glycerol sn1 and sn3 protons. If successful, in a TAG-only extraction, the theoretical FA/glycerol ratio should be 3^39^. The FA profile (in percentage) for saturated (SFA) and unsaturated fatty acids (UFA), both poly- (PUFAs) and monounsaturated fatty acids (MUFA), was estimated by ^1^H NMR^34^. TAG signals resonating from glycerol and FA (terminal methyl group for DLN and MUFAs’ allylic protons for desaturation rate) were quantified from the ^1^H and ^2^H NMR spectra by measuring the ^1^H and ^2^H intensities of selected signals relative to the ^1^H and ^2^H intensities of a pyrazine standard^39^. Fractional synthetic rates (FSR; in % day^−1^) or desaturation rate (% day^−1^) were estimated by dividing these positional TAG ^2^H-enrichments by that of body water. ^2^H-enrichments were calculated after systematic subtraction of the values with 0.015%, taken as the mean background ^2^H-enrichment. If the values were below zero, these were considered as 0.0 for FSR calculation purposes.

The fat samples collected for mRNA abundance analysis were placed in RNA Stand-by Solution (Grisp, Portugal) and kept at −80 °C. RNA extraction was performed with TRI Reagent (Sigma) and NucleoSpin RNA (Macherey-Nagel) Kit. Briefly, the aqueous phase, containing the RNA obtained with the TRI Reagent, was cleaned with the NucleoSpin RNA columns and with DNase digestion at the spin column. The RNA concentration and purity were assessed with NanoDrop 2000 (Thermo Scientific). cDNA synthesis was performed with iScript cDNA Synthesis Kit (Bio-Rad) from 500 ng of total RNA. Real time quantitative PCR was completed using the Sso Advanced SYBR Green Supermix Kit (Bio-Rad) and threshold cycle (Ct) values were generated by the StepOne Software (Applied Biosystems). The temperature cycle protocol for amplification was: 95 °C for 30 s, followed by 45 cycles of two steps: first step of 5 s at 95 °C, second step of 15 s at the primers’ annealing temperature (Table 1). The mRNA relative quantification was determined by the Pfaffl method, taking into consideration the different amplification efficiencies of all genes in all experiments. β-actin from *Limosa limosa* (accession number JF913946) was chosen as the reference (housekeeping) gene to normalize expression levels of targets between different samples. Primers to assess mRNA abundance for fatty acid synthase (*FASN*), NADP-dependent malic enzyme (*ME1*), acetyl-CoA carboxylase (*ACC*) and lipoprotein lipase (*LPL*) were designed as previously reported in^40^ (Table 1). Most of the *de novo* lipid synthesis and modification occurs in the bird’s liver. To avoid euthanizing the animals, we measured the mRNA abundance in the adipose tissue instead, with the underlying assumption that they will both undergo similar changes due to diet.

**Table 1:** List of primers used to determine the enzymatic expression as reported by Lucia *et al*. ^40^.

Gene	Acession number	Primers 5′-3′	Ta
β-actin	JF913946	F: CCAACTGGGATGACATGGAGAAG R: CCAGAGGCATACAGGGACAA	56 °C
Acetyl-CoA carboxylase	JN122328	F: GTCCTCCAAGCCAAGCAATGTG R: GGCCTTGATCATGACAGGGTAGCC	59 °C
Fatty acid synthase	JF913947	F: GCTCCAAAGGCTCTGCG R: AGCACAACAGGCATTTGCTC	55 °C
Lipoprotein lipase	JN122329	F: GCCGTAAGAACCGCTGC R: AGTGCCATAGAGAGAGATCAGG	55 °C
NADP-dependent malic enzyme	JN122330	F: ATCAAGGCTATTGTGGTGACAG R: ATTCTCTTGTGTCTCAGCCC	54 °C

Ta = annealing temperature.

All experimental procedures complied with the guidelines of the European Union (Directive 2010/63/EU) and they were approved by the bioethical committee of the University of Extremadura with permission number 82//2014.

### Statistical analyses

To ensure there was no bias in our experimental set up regarding body mass of birds in each group, we tested body mass differences between groups during the experimental period using repeated-measures analysis of variance (one-way ANOVA). ANOVA was used to test for differences in plasma metabolite levels, TAG composition and enzymatic mRNA abundance between the three study groups. *A posteriori* Tukey’s multiple comparisons test were performed when significant differences were found. In the case of FSR analysis, where signal for the Fasted group was not detected, differences between Rice and Larvae groups were tested using Student’s t-test. Data is presented as mean ± SEM and all statements of significance are based on testing at *P ≤ *0.05. The statistical procedures were performed in R (R Core Team 2013) using several functions within different R packages (psych, doBy, plyr, MASS, lme4, lmerTest, ggplot2), and in GraphPad Prism software (GraphPad Software, La Jolla, CA, USA). Response variables were tested for normality (Q-Q plots) and homogeneity (Cleveland dotplots)^41^.

## Results

### Body condition and plasma metabolites

No significant differences in body mass were observed between godwits from Fasted, Rice and Larvae groups at any stage (Table 2): day of experiment onset (day 1; F _2,19_ = 2.72 P = 0.092); day of intraperitoneal injection (after 48 h of fasting – day 23; F_2,20_ = 0.603; P = 0.557); day of refueling (day 24; F_2,20_ = 2.419; P = 0.115); and day of release (day 29; F_2,20_ = 0.867; P = 0.435). In accordance to the amount of ^2^H_2_O administered intraperitoneally and reinforced with the ^2^H-enriched drinking water, body water ^2^H-enrichment was approximately 5%, with no differences between groups (Table 2).

**Table 2:** Body mass (g) and body water ^2^H-enrichment (%) from black-tailed godwits submitted to three different conditions in captivity (Fasted, Rice and Larvae).

	Fasted (n = 6)	Rice (n = 8)	Larvae (n = 8)
Initial weight (g)	235.77 ± 17.96	281.96 ± 35.24	258.56 ± 47.03
IP injection day weight (g)	226.89 ± 21.13	239.96 ± 27.73	242.38 ± 42.83
Sampling day weight (g)	214.56 ± 21.48	243.89 ± 28.64	249.40 ± 43.48
Release day weight (g)	233.21 ± 20.98	275.34 ± 29.55	259.23 ± 45.09
Body water ^2^H-enrichment (%)	5.3 ± 0.4	5.3 ± 0.3	5.0 ± 0.3

Mean values ± SEM are presented. No significant differences between dietary treatments (one-way ANOVA followed by Tukey’s test).

**Figure 2:**
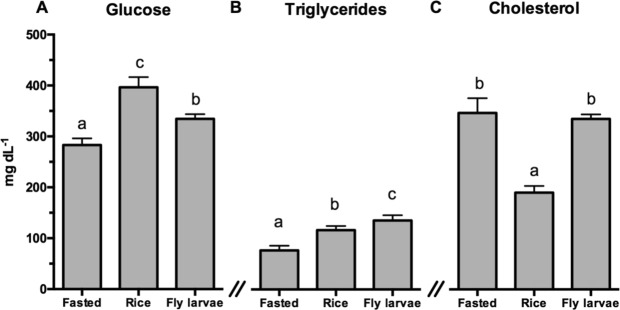
Variation in (**A**) Glucose, (**B**) Triglycerides and (**C**) Cholesterol levels in plasma of black-tailed godwits submitted to three different conditions in captivity (Fasted, Rice and Larvae). Values are presented as mean ± SEM. Significant differences between groups are indicated with different letters (one-way ANOVA with Tukey’s post-hoc test; p < 0.05).

Plasma glucose was significantly lower in the fasted birds than in the Rice and Larvae groups, being highest for the Rice group (F_2,19_ = 15.02; P ≤ 0.0001; Fig. 2A). Plasma triglycerides were also significantly lower in the Fasted (F_2,19_ = 10.48; P < 0.01; Fig. 2B) and in the Rice (F_2,19_ = 10.48; P < 0.01; Fig. 2B) groups than in Larvae group. Plasma cholesterol was significantly lower for the Rice group compared to the two other groups (F_2,19_ = 21.64; P < 0.0001; Fig. 2C).

### TAG composition and synthesis

The FA/glycerol ratio was consistent and approximately 3 for all birds (3.15 ± 0.05) with no significant differences between groups (P < 0.05), as expected from TAG preparations. Despite the possibility of having other neutral lipids in the mixture, it was considered cost-ineffective to further purify lipid samples if they were to be analyzed with relatively low sensitivity instrumentation such as NMR (in comparison to mass-based instrumentation). TAG composition differed in terms of total SFA, UFA and MUFA among groups, with the Rice group having lower UFA and MUFA, but higher SFA, than the other two groups (Table 3). Furthermore, ^1^H NMR spectra of TAG from both Rice and Larvae groups had quantifiable n-3 FA, although significantly lower levels for the Larvae group (Table 3).

**Table 3:** Lipid species and chemical structure of esterified fatty acids as determined from ^1^H NMR spectra of subcutaneous fat triglycerides from black-tailed godwits submitted to three different conditions in captivity (Fasted, Rice and Larvae).

Lipid species (%)	Fasted (n = 7)	Rice (n = 7)	Larvae (n = 8)
% SFA	20.5 ± 5.1^a^	34.7 ± 3.1^b^	21.5 ± 0.8^a^
% UFA	79.6 ±± 5.1^a^	65.3 ± 3.1^b^	78.5 ± 0.8^a^
% PUFA	13.7 ± 2.5^a^	14.4 ± 1.4^a^	10.5 ± 0.6^a^
% MUFA	65.8 ± 2.8^a^	50.9 ± 2.5^b^	68 ± 0.6^a^
% n-3	5.6 ± 1.8^a^	1.9 ± 0.6^ab^	1.2 ± 0.5^b^

Mean values ± SEM are presented. Significant differences between dietary treatments are indicated by different letters (one-way ANOVA, p < 0.05; followed by Tukey’s test).

**Figure 3:**
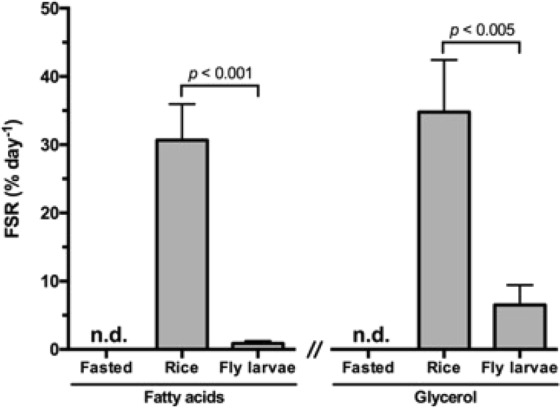
Triglyceride-bound fatty acid and glycerol fractional synthetic rates (FSR) expressed as percent per day of subcutaneous fat triglycerides from black-tailed godwits submitted to three different diets (Fasted, Rice and Larvae) and to ^2^H_2_O administration for 24 h. Mean values ± SEM are presented (n = 7 for Fasted and Rice; n = 8 for Larvae). No labelling detected in fasted birds: n.d. Differences between dietary treatments are indicated (t-test, p < 0.05).

After 24 hours of the refueling period, we observed significant differences in *DNL* activity between Rice and Larvae groups, with a significantly higher rate of fractional *DNL* in the Rice group (30.7 vs. 0.9% day^−1^, respectively; p < 0.0001; Fig. 3). This was also accompanied by increased desaturation rates compared to the Larvae group (14.7 vs. 0.2% day^−1^; p < 0.0001), meaning that nearly half of the newly-synthesized FA were also desaturated. With regards to glycerol, ^2^H-enrichment was also higher in the Rice group with values very similar to those reported for FA of the same group (34.8% day^−1^_,_ Fig. 3). The glycerol ^2^H-enrichment in Larvae group was considerably higher relative to the observed FA-enrichments in the same group (6.5% day^−1^, Fig. 3). Enrichment derived from ^2^H_2_O in TAG and glycerol of birds in the Fasted group was not detected, as expected in a scenario of null lipogenic activity.

### Enzymatic mRNA abundance

Despite our best efforts we were unable to extract mRNA from 8 of the 23 samples. Sampling from the Fasted group was particularly difficult due to very low amounts of visible adipose tissue. Another two samples gave extremely discrepant Ct duplicate readings in every run and gene assay. These were removed from the analysis resulting in small sample sizes: Fasted: n = 3; Rice n = 5; Larvae n = 5.

**Figure 4:**
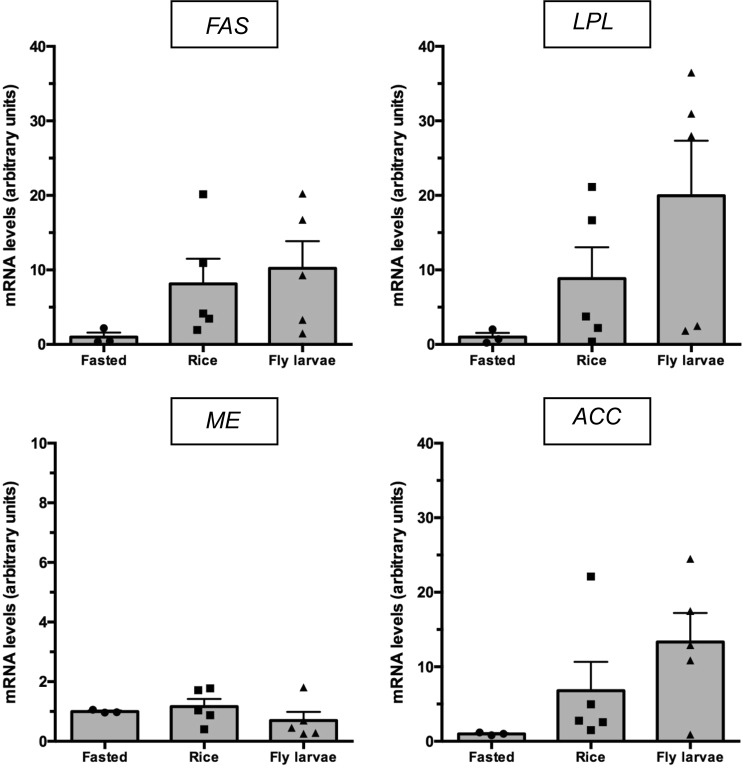
Fatty acid synthase (*FASN*), NADP-dependent malic enzyme (*ME1*), acetyl-CoA carboxylase (*ACC*) and lipoprotein lipase *(LPL)* mRNA abundance in subcutaneous fat from black-tailed godwits submitted to three different diets (Fasted, Rice and Larvae). Values are presented as mean ± SEM (n = 3 for Fasted, n = 5 for Rice and Larvae). One-way ANOVA tested indicated no significant differences.

Despite an overall lower mRNA abundance for the tested genes in the Fasted group, this was not significant in any of the tested lipogenic genes (Fig. 4). This may be attributed to the low number of samples but also the high level of variance observed in the response of such enzymes in the Rice and Larvae groups’ mRNA abundance levels (Fig. 4). An exception was *ME1* (NADP-dependent malic enzyme) which presented similar levels in all three groups.

## Discussion

We assessed the dietary regulation of lipid synthesis in a long-distance migrating shorebird during a fasting and refueling cycle, when feeding on contrasting diets, mimicking dietary changes documented for this species in recent years. As expected, staging godwits that fed on rice seeds were able to replenish their fat reserves by substantially increasing *DNL* synthesis, thus compensating for a lower dietary FA content. This confirms the results by Viegas *et al*.^34^ attained for the wintering period and extends such metabolic plasticity to a more demanding period of the annual cycle, such as migration: the refueling phase in the stopover site after a fasting period. The higher glycerol synthesis found in godwits that feed on rice may indicate that fattening rates observed at this group was higher. This meaning that migratory birds that feed on carbohydrates can benefit by increasing their fat reserves faster than birds that feed on lipid diets. Refueling at stopover sites after a fasting period of flight is a typical cycle in migrating birds. It should be noted that the induced fasting period fails to include the demanding exercise of flying which is an integral part of migration. But when in conjunction (fasting and exercise), acute lipogenic activity might be triggered - a hypothesis that requires further testing. According to Robin *et al*.^42^, the signal inducing re-feeding activities is triggered in spontaneously fasting birds during migration, if the birds still have fat reserves available. Physiological adjustments occur gradually^43–45^, and godwits can be a good study case to understand the effects of nutritional shifts and trigger signals in migratory birds.

Our results also indicate that a rice diet can provide a profitable trophic resource due the high *DNL* synthesis observed in rice group and with the already described ecological advantages (e.g. predation, disturbance)^10,46,47^ make this habitat important for shorebirds and other migratory waterbird species that use rice fields during spring migration.

### Body condition and plasma metabolites

Birds present significantly higher blood glucose concentrations than other vertebrates with similar body mass^48–50^. In general, birds use glucose for a wide variety of functions such as energy production through cellular oxidation and FA synthesis^43^. Despite the absence of any food intake, godwits in the fasted group also had high glucose levels, demonstrating that birds can sustain high indices of glucose in plasma. The main reason is that glucose is apparently not metabolized to sustain long migratory flight, nor during fasting periods^48,51^. For example, semipalmated sandpiper (*Calidris pusilla*) feeding on the amphipod *Corophium volutator*, i.e. a lipid-rich diet, can fly non-stop between the breeding and wintering grounds, whilst showing high plasma glucose concentrations on arrival and on departure^19^. The lack of differences in blood glucose levels between non-migratory and migratory bar-tailed godwits (*Limosa lapponica taymyrensis*) also suggest no glucose metabolization during long non-stop flights^51^. Therefore, overall, high glucose concentrations were likely maintained as a result of migratory birds primarily using FA to fuel metabolism^45^. Likely due to the carbohydrate-rich diet, godwits from Rice group showed higher levels of glucose in plasma than godwits from the Larvae and Fasted groups.

Numerous studies measured plasma metabolites to assess the effects of fasting and feeding conditions^52–54^. Generally, plasma triglycerides levels are an excellent indicator of fat metabolism in birds^19,55,56^ as observed in Rice and Larvae groups. In long-term fasting birds, most of the energy requirements are met by lipids^57^. Low levels of triglycerides are associated with short-term fasting in birds^54^, as observed in godwits from the Fasted group. Conversely, godwits from the Rice and Larvae groups showed higher triglyceride levels likely due to the refueling activity, as plasma triglyceride level tends to increase rapidly with feeding activities or fattening processes^58^. However, Smith and McWilliams^56^ demonstrated that differences in diet can affect indices of fat metabolism and subsequent body fat storage. The increase in triglycerides depends primarily on lipid availability both from the diet and from *de novo* lipogenesis^50^. Poultry studies indicated that protein-rich diets (e.g. fly larvae) condition the fattening process by inhibiting the novo lipogenesis^59–61^. In contrast, low protein diets generally result in higher levels of plasma triglycerides. Therefore, godwits fed on rice seeds likely showed high triglyceride levels due to their higher *DNL* rates, and godwits fed on fly larvae also showed high levels of plasma triglycerides, but due to their recent feeding activity and lipid-rich diet. Supporting our findings, a previous study showed in yellow-rumped warblers that carbohydrate-rich diets were associated with higher triglyceride levels during flight^54^. Furthermore, Smith *et al*.^62^ observed in a migratory songbird, the white-throated sparrow (*Zonotrichia albicollis*), that after an overnight fast different diet compositions had no effect on lipid metabolites. If plasma metabolites directly reflect diet composition, we would expect higher plasma triglycerides levels on godwits that feed on larvae (protein-rich diet), than on godwits that feed on rice (carbohydrate-rich diet), as observed.

An increase in plasma cholesterol can be correlated with fattening periods and has been reported to be associated with improvement in the mobilization of dietary fat^63,64^. Cholesterol, as an anabolic precursor to bile acids, could play a determining role in maximizing dietary fat digestion, absorption, and consequently its accumulation. Since godwits in the Rice group endogenously synthesised the majority of their accumulated FA, cholesterol levels were significantly lower when compared to both Fasted and Larvae groups. However, Lehninger^65^ suggested that birds fed on a low-protein diet increase their cholesterol levels in plasma by decreasing the excretion of cholesterol in the form of bile acids. The high cholesterol levels observed in migratory rosy starling (*Sturnus roseus*) was reported as resulting from the intense pre-migratory feeding activity, stimulating fat deposition through an increase of dietary fat absorption^66^. The re-feeding activity in our godwits, after 48 h of fasting, can explain the higher plasma cholesterol in the Larvae group as some studies demonstrated that cholesterol remained stable during fasting and during migration^64,67–69^. Totzke *et al*.^64^ reported a stable pattern in cholesterol levels in fasting gulls, but the cholesterol levels can be influenced by previous diet. In our case, the higher cholesterol levels in Fasted group can be related with the previous diet, a carbohydrate-rich diet. Moreover, cholesterol levels increased in food-deprived buzzards (*Buteo buteo*) in relation to the lower values observed in fed states^70^. This suggests that birds re-fed recently on a protein-rich diet and birds that feed on low protein-rich diet before a fasting period can show higher cholesterol levels than birds that re-feed on a carbohydrate-rich diet. It is hard to explain this result because we expected high levels of cholesterol synthesis in both re-feeding groups (Rice and Larvae groups). The higher levels of cholesterol in fasted godwits may be due to a physiological degradation of corporal lipidic components that contain cholesterol enhancing the cholesterol in plasma^70^.

### Adipose lipid composition

Fasting godwits showed both low *DNL* and glycerol synthesis, as well as lower levels of triglycerides in plasma. This suggests that during fasting, birds suspend anabolic pathways. During fasting periods, fuel reserves decrease by mobilization and oxidization of FA from TAG, and for this reason, the total of stored TAG decreases. In contrast, structural lipids (phospholipids) should not decrease during fasting. Fasting godwits showed higher levels of omega-3 PUFAs mainly because the cells need to maintain their phospholipid structures to remain functional. When fasting godwits’ TAG stores become depleted, given that SFA are metabolized first, the proportion of PUFAs in the phospholipids will increase. Birds from the Rice group showed a similar level of n-3 PUFAs to those from Larvae group. This may indicate that due to its importance, even under a lipid-poor diet, godwits refuelling on rice seeds likely must prioritize their muscular function by high levels of *de novo* lipogenesis to conserve membrane integrity, thereby conserving the levels of n-3 PUFAs in their fat stores^15^. Similar to semipalmated sandpiper that converted n-3 PUFAs into other FA, such as oleic acid (UFA^14^), our results show that godwits refuelling on fly larvae display low levels of n-3 PUFAs but high levels of UFAs, adopting a similar strategy in order to increase FA stores. Moreover, godwits refuelling on rice had higher levels of SFAs than those in Larvae and Fasted groups, likely due to higher *DNL*. Larvae group godwits presented higher UFA, and this can indicate that during fasting they do not mobilize all the UFA. As shorter and unsaturated FAs are usually mobilized preferentially during fasting periods^71^, we expected that both Larvae and Rice feed groups would present fat stores with more saturated and long chain FA in their fat stores. The higher values of UFA in the Larvae group results probably from a lower *DNL* and new synthesis of glycerol.

### Enzymatic mRNA abundance

Egeler *et al*.^72^ showed upregulation of lipogenic enzymes at stopover sites in Western sandpipers (*Calidris mauri*). Despite the small sample size, we found no significant differences in fat stores regarding enzymatic expression for *ACC*, *LPL*, *ME1* and *FASN* gene expression. We expected that the Rice group would show higher enzymatic expression for *FASN* and *ME1* due to the increase in *DNL*, which directly depends on *FASN* and *ME1* activity. *ME1* is involved in the transport of Acetyl-CoA to the cytosol (citrate shuttle), which promotes the synthesis of NADPH, an important coenzyme for the *DNL* process. *ACC* is an anabolic enzyme that promotes the synthesis of malonyl-CoA for *DNL*. Hermier^73^ stated that *LPL* seems to be less sensitive, and less responsive to changes in nutritional state, in birds’ adipose tissue than in muscle. We expected lower *LPL* expression in the Larvae group due to its lower *DNL* activity, but observed the opposite. Furthermore, *DNL* is highly dependent on a carbohydrate-rich diet providing Acetyl-CoA to stimulate activity of the main enzymes involved, such as *ME1* and *FASN*. The latter can be rate-limiting, especially when a bird is in negative balance of energy^72^, such as when fasting. Re-feeding birds in a high-carbohydrate, low-fat diet, should cause an increase in *ACC*^74^ and high levels of *DNL*. However, contrary to our expectations, we observed a trend towards a lower level of *ACC* in the Rice group when compared with birds that were re-fed on larvae.

## Conclusions

The migratory species used as model in this study revealed the capacity to adapt its lipid metabolism and compensate for a poorer dietary lipid content (carbohydrate-rich diet) by considerably increasing *DNL* levels. This species can change its metabolic patterns according to the environmental challenges faced during the migratory cycle, mainly during the stopover periods. Studying the responses of species to dietary constraints will contribute towards predictions of how species will adapt to a fast-changing environment.
